# A Retrospective Tribute to Dr. Harish Pant (1938–2023) and His Seminal Work on Cyclin Dependent Kinase 5

**DOI:** 10.1007/s11064-024-04234-5

**Published:** 2024-09-05

**Authors:** Bradford Hall, Niranjana Amin, Veeranna, Shin-ichi Hisanaga, Ashok. B. Kulkarni

**Affiliations:** 1 Functional Genomics Section, National Institute of Dental and Craniofacial Research, Bethesda, MD 20892 USA; 2 Cytoskeletal Protein Regulation Section, National Institute of Neurological Disorders and Stroke, National Institutes and Health, Bethesda, MD 20892 USA; 3 Laboratory of Molecular Neuroscience, Department of Biological Sciences, Graduate School of Science, Tokyo Metropolitan University, Minami-Osawa, Hachioji, Tokyo 192-0397 Japan

**Keywords:** Cyclin-dependent kinase, Neurofilament, Alzheimer disease, Neurons, Pain

## Abstract

Dr. Harish Chandra Pant was Chief of the Section on Neuronal Cytoskeletal Protein Regulation within the National Institute of Neurological Disorders and Stroke at the NIH. A main focus of his group was understanding the mechanisms regulating neuronal cytoskeletal phosphorylation. Phosphorylation of neurofilaments can increase filament stability and confer resistance to proteolysis, but aberrant hyperphosphorylation of neurofilaments can be found in the neurofibrillary tangles that are seen with neurodegenerative diseases like Alzheimer disease (AD). Through his work, Harish would inevitably come across cyclin dependent kinase 5 (Cdk5), a key kinase that can phosphorylate neurofilaments at KSPXK motifs. Cdk5 differs from other Cdks in that its activity is mainly in post-mitotic neurons rather than being involved in the cell cycle in dividing cells. With continued interest in Cdk5, Harish and his group were instrumental in identifying important roles for this neuronal kinase in not only neuronal cytoskeleton phosphorylation but also in neuronal development, synaptogenesis, and neuronal survival. Here, we review the accomplishments of Harish in characterizing the functions of Cdk5 and its involvement in neuronal health and disease.

In the early 90’s, the hunt was on to identify new kinases that were related to cell cycle-associated cyclin dependent kinases (Cdks) but could also phosphorylate neuronal proteins such as tau and neurofilaments in post-mitotic neurons. Using primers that correspond to conserved regions of *cdc2*, Dr. Li-Huei Tsai’s group was able to amplify out cdc2-related protein kinases from human cell lines. One of these was designated PSSALRE kinase, which was found to be highly expressed in the adult brain [1]. Dr. Jerry Wang’s lab was also trying to identify a cdc2-like family member that was not involved in cell division but could regulate cellular processes in differentiated neurons in the brain. Eventually, they purified a proline-directed protein kinase from bovine brain [2]. Around the same time, Dr. Harish Pant was collaborating with Dr. James F. Battey’s lab at NCI to screen a rat brain cDNA library and identify a neuronal cdc2-like kinase that would phosphorylate KSPXK motifs in neurofilaments [3]. The cdc2-like kinase identified by these groups (PSSALRE, brain proline-directed kinase, and neuronal Cdc2 like kinase) was later designated cyclin dependent kinase 5 (Cdk5) [4]. Cdk5 was concurrently identified as tau protein kinase II, linking Cdk5 to the paired helical filaments that accumulated in the brains of Alzheimer’s disease (AD) [5]. An interest in Cdk5 and its role in neurodegenerative diseases like AD would essentially from then on became a major focus of Harish Pant’s research.

**Figure:**
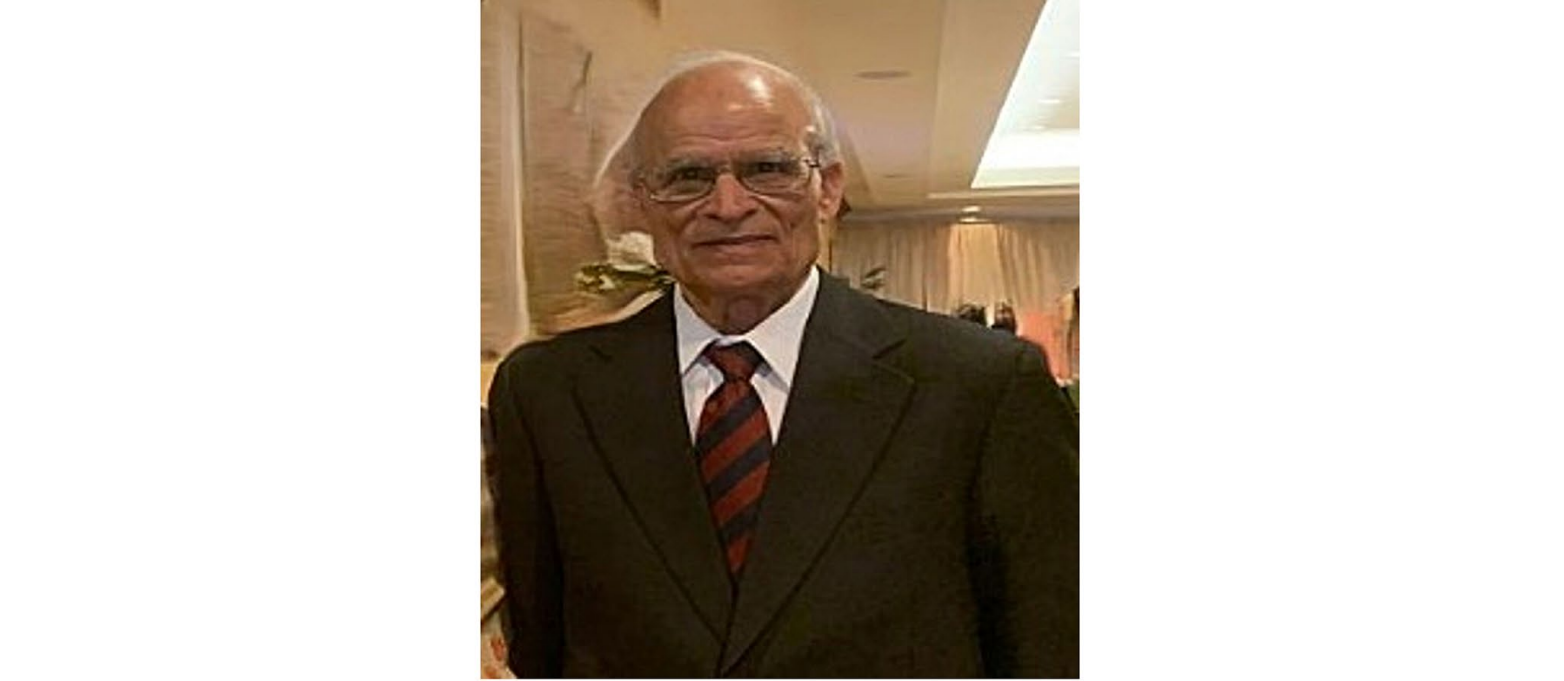

Dr. Harish Pant was born in Jakhera town in Pithoragarh District of Uttarakhand state in India and got his Ph.D. degree in Physics from Agra University. He started working at the NIH in 1974 and later joined the National Institute of Neurological Disorders and Stroke (NINDS) within the Laboratory of Neurochemistry. Eventually, he led the Neuronal Cytoskeletal Protein Regulation Section. Initial work centered on understanding the functions of neuronal cytoskeleton proteins, some of which involved the giant squid axon model system [6–8]. It was through the work on neurofilaments (NF) that Harish got involved in Cdk5. Neurofilaments are intermediate filaments within the cytoskeleton that are the most abundant components within neuronal axons [9]. Phosphorylation of neurofilaments NF-M (medium chain) and NF-H (heavy chain) controls interactions with microtubules, which can then affect axonal transport [10, 11]. Hyperphosphorylated neurofilaments are also components of neurofibrillary tangles that are commonly detected in patients with neurodegenerative disorders such as amyotrophic lateral sclerosis (ALS), Parkinson’s disease (PD), and Alzheimer disease (AD) [9]. Harish’s lab was able to identify several KSPXK repeats (a typical motif for Cdks) within the carboxy-terminal tail domain of NF-M/H that could be phosphorylated by Cdk5 [12–14]. Significantly, the KSPXK motifs within NF-M/H were hyperphosphorylated in the brains of AD patients, which further tied Cdk5 with neurodegenerative diseases [9]. Harish’s group alternately identified Protein Phosphatase 2 as the enzyme that could then dephosphorylate NF-H [15]. It is also worth mentioning that Cdk5 can be found in multimeric complexes that includes neuronal cytoskeletal proteins along with other kinases [16]. That is because cytoskeletal proteins can require phosphorylation by multiple different kinases. In particular, Harish’s lab demonstrated that Cdk5 can be complexed with cytoskeletal proteins and Erk1/2, another proline-directed protein kinase that is fundamental in phosphorylating KSP motifs in the tail domains of neurofilaments [16–18]. Along with neurofilaments, Cdk5 can also phosphorylate the microtubule-associated protein tau, with hyperphosphorylated tau being detected in neurofibrillary tangles as well [19]. Despite initially being linked with neuropathological diseases, Cdk5 was later found to have an important physiological role in modulating neuronal activity such as regulating synaptic plasticity as well as neuronal exocytosis/endocytosis [20, 21]. This was particularly apparent as targeted deletion of Cdk5 in mice results in perinatal lethality and defects in corticogenesis [22]. With the knockout mice, it became clear that Cdk5 has a role in neuronal migration and axonal guidance during brain development, yet Cdk5 activity has also been detected in the peripheral nervous system where it is important in regulating pain signaling [23, 24].

Cdk5, like other Cdks, is not activated by second messenger molecules like cAMP or Ca^2+^, but instead requires binding with a regulatory protein subunit [25]. Cdk5 is primarily activated by either p35 or p39. Both activators p35 and p39 are distinct from the cyclins that activate other Cdks, but they do structurally possess a cyclin-box fold topology that is needed for Cdk5 activity [26]. The expression pattern of p35 and p39 are known to limit Cdk5 activity primarily to post-mitotic neurons [20]. Of the two, the regulatory subunit p35 is the better characterized activator, especially as the Cdk5/p39 complex is less stable than Cdk5/p35 [27]. To better understand the kinase activity of Cdk5/p35, Harish’s lab was able to map out three Cdk5 binding domains with p35 [28] while also modeling the Cdk5/p35 substrate binding site [14]. His group also demonstrated that Cdk5 requires both p35 binding together with phosphorylation at Ser^159^ within its activation loop or T-loop for maximal rates of activation [29].

With excess Ca^2+^ influx within the neuron, such as during neuronal excitotoxicity, the calcium-activated protease calpain is activated to cleave the Cdk5 activator p35 into the p25 and p10 [30]. Although cleaved, the resulting truncated p25 is still capable of binding and activating Cdk5 while having a longer half-life than p35 by about 5- to 10-fold [31, 32]. Normally, p35 is myristoylated and Cdk5 is thereby tethered to the plasma membrane, but after being cleaved, Cdk5/p25 then becomes mislocalized within the cytosol to essentially hyperphosphorylate cytoskeletal proteins like tau and NF-M/H. Cdk5/p25 complexes can subsequently cause neuronal death, where notably the accumulation of p25 has been detected in the brains of AD patients [33]. Hence, Cdk5/p35 is considered to be important for normal physiological functions in neurons while Cdk5/25 is more characterized in pathological conditions [32]. A key therapeutic strategy to treat neurodegenerative diseases would thereby involve finding a means to interfere with the pathological activity of Cdk5 by blocking its interaction with p25 while still maintaining its normal neuronal functions when complexed to p35. This was a challenge Harish Pant’s group took on by designing inhibitory peptides to specifically block the binding of p25 to Cdk5. While mapping the interacting domains within p35 that are required for Cdk5 binding, a short peptide (amino acid residues 154–279) was identified that had a high affinity for Cdk5 yet did not induce kinase activity [28]. This Cdk5 inhibitory peptide (CIP) interestingly blocks the pathological p25-mediated activities of Cdk5 without interfering with its ‘normal’ physiological kinase activity [34]. CIP also does not interfere with the activity of other Cdks. This is of concern as small molecule inhibitors designed to compete for the Cdk5 ATP binding site often tend to non-specifically inhibit other Cdks as well. Further testing demonstrated that CIP could prevent Cdk5/p25 mediated phosphorylation of tau and inhibited amyloid Aβ induced apoptosis [34, 35]. Additionally, CIP was shown to be neuroprotective in a mouse model of ALS [36]. However, CIP, at 125 a.a., was too large to be therapeutically useful, so Harish’s lab identified a shorter 24 a.a. version of the inhibitory peptide, named p5 (245–277), that had even better efficacy in blocking Cdk5/p25 hyperactivity than CIP [37]. A transactivator of transcription (Tat) peptide was later added to create TP5 so that the inhibitory peptide could be more readily transported across the blood-brain barrier. TP5 could then be injected intraperitoneally where it was shown to be neuroprotective in the 5XFAD mouse model of AD and in the MPTP model of PD [38–41]. So, a therapeutic such as TP5 would be preferable in that it is more selective and only targets Cdk5/p25 hyperactivity without interfering with normal cellular Cdk activity, including Cdk5/p35 [37].

As described, research into Cdk5 and its role in neurodegenerative diseases were a major part of Harish’s research, but Cdk5 can be characterized as a ‘two-faced’ kinase in that its normal physiological activity is also important for neuronal survival [42]. In this regard, Harish’s lab was able to identify crosstalk between Cdk5 and other neuronal signaling pathways to show that Cdk5 activity has an important role in regulating neuronal development. Firstly, Cdk5 can regulate the mitogen-activated protein kinase pathway by inhibiting activated MEK1 signaling via phosphorylation of Thr^286^ within the proline-rich domain. Phosphorylation of MEK1 prevents the neuronal apoptosis that can result from sustained downstream Erk1/2 activation [42, 43]. Besides phosphorylating MEK1, Cdk5 can modulate Erk1/2 activity at an upstream growth factor signaling control point through phosphorylation of RasGRF2 on Ser^737^ [44]. Phosphorylation of RasGRF decreases subsequent Rac–guanidine exchange factor activity leading to a decrease in the levels of activated phospho-Erk1/2. Cdk5 may alternatively promote neuronal survival by phosphorylating c-Jun N-terminal kinase 3 (JNK3) on Thr^131^ to negatively impacts its activity [45]. JNK3 is considered a stress-activated protein kinase that promotes neuronal apoptosis, where the lack of Cdk5-mediated inhibition of JNK3 may partly account for the higher numbers of apoptotic neurons in Cdk5 knockout mice as well as the increased sensitivity to UV-induced apoptosis in Cdk5^−/−^ cultured cortical neurons. Besides the higher JNK3 activity in Cdk5^−/−^ cultured cortical neurons, there is also lower phosphatidylinositol 3-kinase (PI3K) and phosphorylation of Akt, both of which are key in mediating neuronal survival [46]. In this case, Cdk5 is thought to regulate the PI3K/Akt signaling pathway by phosphorylating the neuregulin receptors (ErbB2/ ErbB3) to protect against apoptosis. Lastly, neural stem cells (NSCs) derived from E13 Cdk5^−/−^ embryos show reduced neuronal differentiation. Cdk5 can phosphorylate p27^Kip1^ [47], a Cdk inhibitor that is involved neurogenesis, where Harish’s lab determined that Cdk5 regulates neural differentiation via the phosphorylation of theThr^187^ site in p27^Kip1^ [48]. So, although originally identified as a kinase that could be involved in neurofibrillary tangles and neurodegeneration [3, 5], Cdk5 was later determined to be important for neuronal survival and differentiation.

The importance of regulating neuronal activity such as synaptic plasticity and neuronal growth via protein phosphorylation has long been understood [49]. So, the identification of Cdk5 as a kinase that is mainly active in post-mitotic neurons has, not surprisingly, drawn the interest of many researchers, especially with Cdk5 also being linked to the neurofibrillary tangles seen in neurodegenerative diseases. Harish certainly hasn’t been the only investigator to focus this unusual kinase, but his work has nonetheless made major contributions to understanding Cdk5’s physiological as well as pathological activities. Harish’s lab studied Cdk5’s role in neuronal survival as well as apoptosis while also attempting to identify novel means of inhibiting aberrant Cdk5 hyperactivity without not interfering with its normal neuronal functions. Along the way, he was also considered a great mentor and helped many scientists further their careers. While more papers could have been cited here and many more words could be said, we hope this overview of Harish’s contribution to identification and characterization of kinases and their important role in neuronal functions provides an idea about his impact to the field of neuroscience, which should be noted and remembered.

Our groups had a distinct honor and privilege to collaborate with Harish’s group on many aspects of Cdk5 research. Together, we in Kulkarni Lab characterized molecular roles of Cdk5, and its activators p35 and p39 in numerous neuronal functions that impact important physiological functions. We also worked with Harish’s group on understanding the role of Cdk5 in corticogenesis [22], and, recently, in identifying Cdk5 as a key player in pain signaling, especially since chronic pain affects 20% of adults in the United States and has an annual cost to society of roughly $635 billion dollars, exceeding the costs of cancer, heart disease, and diabetes combined [50].

## Data Availability

No datasets were generated or analysed during the current study.
